# Imputing accelerometer nonwear time in children influences estimates of sedentary time and its associations with cardiometabolic risk

**DOI:** 10.1186/s12966-019-0770-0

**Published:** 2019-01-17

**Authors:** M. M. Borghese, E. Borgundvaag, M. A. McIsaac, I. Janssen

**Affiliations:** 1 School of Kinesiology and Health Studies, Queen’s University, 28 Division St, Kingston, ON Canada; 2 Department of Public Health Sciences, Queen’s University, 99 University Avenue, Kingston, Ontario Canada

**Keywords:** Missing data, Multiple imputation, Accelerometer, Sedentary behaviour, Child, Health

## Abstract

**Background:**

A limitation of measuring sedentary time with an accelerometer is device removal. The resulting nonwear time is typically deleted from the data prior to calculating sedentary time. This could impact estimates of sedentary time and its associations with health indicators. We evaluated whether using multiple imputation to replace nonwear accelerometer epochs influences such estimates in children.

**Methods:**

452 children (50% male) aged 10–13 were tasked with wearing an accelerometer (15 s epochs) for 7 days. On average, 8% of waking time was classified as nonwear time. Sedentary time was derived from a “nonimputed” dataset using the typical approach of deleting epochs that occurred during nonwear time, as well as from an “imputed” dataset. In the imputed dataset, each nonwear epoch was re-classified as being as sedentary or not using multiple imputation (5 iterations) which was informed by the likelihood of a wear time epoch being classified as sedentary or not using parameter estimates from a logistic regression model. Estimates of sedentary time and associations between sedentary time and health indicators (cardiometabolic risk factor and internalizing mental health symptoms Z-scores) were compared between the nonimputed and imputed datasets.

**Results:**

On average, sedentary time was 33 min/day higher in the imputed dataset than in the nonimputed dataset (632 vs. 599 min/day). The association between sedentary time and the cardiometabolic risk factor Z-score was stronger in the imputed vs. the nonimputed dataset (β = 0.137 vs. β = 0.092 per 60 min/day change, respectively). These findings were more pronounced among children who had < 7 days with ≥10 h of wear time.

**Conclusion:**

Researchers should consider using multiple imputation to address accelerometer nonwear time, rather than deleting it, in order to derive more unbiased estimates of sedentary time and its associations with health indicators.

## Introduction

There is evidence, albeit mixed, that objectively-measured sedentary time is associated with health indicators during childhood [1–4]. School-aged children accumulate ≈8.5 h/day of sedentary time [5, 6] and their sedentary behaviour patterns track into adulthood [7, 8] where there is some evidence that excessive sedentary time increases the risk of chronic disease and mortality [9–11]. Therefore, studying sedentary time in children is important and the accurate measurement of this construct is of significance to sedentary behaviour research.

Accelerometers are commonly used to measure sedentary time. One of the problems encountered when measuring sedentary time with an accelerometer is “nonwear time”. Nonwear time occurs when a participant removes their accelerometer during the measurement period. Participants do this for a variety of reasons: they may not like the way the accelerometer looks, it may be uncomfortable to wear, or they may remove it when it could get wet [12, 13]. The vast majority of child participants have some nonwear time during a typical one-week accelerometer measurement period [6, 14–16].

Processing of raw accelerometer data typically consists of deleting all epochs collected during nonwear time, all epochs that occurred on days with too much nonwear time (e.g., < 10 waking hours of wear time [15, 17, 18]), and all data from participants with too many days with too much nonwear time (e.g., > 3/7 days with < 10 h of wear time) [15, 17, 18]. There are three problems with this processing approach. First, estimates of sedentary time in minutes/day are likely underestimated because they are calculated from incomplete accelerometer data. However, the direction of bias is not known for estimates of the proportion of wear time spent sedentary; this depends on whether children are more or less sedentary during nonwear time. It has been suggested that the proportion of time spent sedentary might be greater during nonwear time compared to wear time [19]. Second, estimates of sedentary time cannot be derived for participants that are excluded because they have excessive nonwear time; this typically represents 15–30% of child participants [6, 15, 16, 20]. The exclusion of these participants could bias group-level estimates of sedentary time and the association between sedentary time and health indicators if these participants are systematically different from the participants who were not excluded. Third, removing participants because of excessive nonwear time can substantially reduce statistical power and precision.

The three aforementioned problems could potentially be addressed by imputing nonwear accelerometer epochs with plausible values rather than deleting them during accelerometer data processing. Multiple imputation should be used to capture the uncertainty in the imputation of the unobserved data in order to allow for appropriate variance estimation [21–23]. Lee and Gill (2016) employed a multiple imputation approach that used a zero-inflated Poisson log-normal model to account for the zero-inflated distribution and autocorrelated structure of the accelerometer epoch count data [23]. The authors showed that this model predicted missing accelerometer count data with greater precision (using observed count values as the reference) than three other comparative models. However, they did not compare summary estimates of sedentary time, or associations between sedentary time and health risk, between the nonimputed vs. imputed datasets. Thus, the impact that an imputation approach has on estimates of sedentary time (both in minutes/day and the proportion of wear time spent sedentary) and its associations with health risk remains unknown. Recently, Paul and McIsaac explored the utility of different imputation approaches [24], including those developed by Lee and Gill (2016), in both predicting missing accelerometer count data and in deriving summary estimates of moderate-to-vigorous physical activity. They demonstrated that simpler imputation approaches are adequate for the purpose of deriving summary estimates and that more complicated approaches add little value in reducing bias in summary estimates. Thus, deriving and comparing summary estimates of sedentary time should allow for the selection a relatively simple imputation approach, such as imputing a binary variable.

Our lab group recently developed and implemented a relatively simple (i.e., binary) multiple imputation approach for nonwear accelerometer epochs of a moderate-to-vigorous intensity [25]. The model used in our approach included several time-based, socio-demographic, and health-related correlates of physical activity [25]. We showed that using multiple imputation did not influence estimates of time spent in moderate-to-vigorous physical activity or associations between moderate-to-vigorous physical activity and cardiometabolic risk factors. It is possible that this imputation approach would have a greater impact on estimates of sedentary time because it accounts for a much greater proportion of a child’s time [6]. For example, in the average 6–17 year old Canadian, sedentary time is almost 10-fold greater than time spent in moderate-to-vigorous physical activity (510 vs. 55 min/day) [5, 26]. Moreover, this imputation might have an impact on the proportion of time spent sedentary, especially since this may be higher during nonwear time compared to wear time [19].

Accordingly, the objective of this paper was to evaluate whether using multiple imputation to replace nonwear accelerometer epochs influences: 1) estimates of mean sedentary time in minutes/day as well as the proportion of wear time spent sedentary, and 2) the association between sedentary time and selected health indicators.

## Methods

### Study participants and data collection overview

A sample of 452 children aged 10–13 years was recruited from Kingston, Ontario, Canada. Data collection occurred between January 2015 and December 2016 and was balanced across the four seasons. An equal number of boys and girls and of 10, 11, 12, and 13 year olds were recruited. Written informed assent and consent was obtained from child participants and their parents/guardians, respectively. This study was approved by the General Research Ethics Board at Queen’s University.

Participants visited the Physical Activity Epidemiology lab at Queen’s University where physical measurements (described below) were obtained. They were instructed to wear an Actical accelerometer (Philips Respironics, Bend, OR) on their right hip for 24 h/day for 7 consecutive days. The devices recorded data in 15 s epochs starting at midnight on the first measurement day. Participants were instructed to remove the device only for aquatic activities (e.g., swimming, showering). Participants recorded the times that they removed their accelerometer, their sleep and wake times, and dates/times that they participated in summer day camps on a log during the measurement period. These times were manually verified, through visual inspection of accelerometer epochs, by the research team with excellent intra- and inter-rater reliability [27]. Participants were sent daily text messages or e-mails to remind them to wear their device and complete their log. Participants were compensated $40 for participating in the study, including $20 for returning the accelerometer in working condition and completing the log.

### Measurement of health indicators

We felt that it was important to include indicators of both physical and mental health to provide a holistic lens of health. The indicator of physical health used in this paper was a cardiometabolic risk factor Z-score. Standing and sitting height were measured to the nearest 0.1 cm using a portable stadiometer (SECA model 213, Hamburg, Germany). Body mass was measured to the nearest 0.1 kg using an electronic scale (Tanita BF-689, Arlington Heights, USA). Body mass index Z-scores were calculated using the World Health Organization age- and sex-specific growth references [28]. Systolic blood pressure and resting heart rate were measured 6 times following 5 min of quiet sitting using a BpTRU BPM-200 (Bayside Medical Supplies Inc., Hawkestone, Canada). The average of the last five measures was used and Z-scores were created. The cardiometabolic risk factor Z-score was created by averaging the Z-scores for body mass index, systolic blood pressure, and resting heart rate.

The indicator of mental health used in this paper was an internalizing symptom score based on 7 questionnaire items that assessed symptoms of anxiety and depression [29, 30]. The questionnaire asked participants to indicate how much they agreed with the following statements: “I am unhappy or sad”, “I am not as happy as other people my age”, “I am too fearful or nervous”, “I worry a lot”, “I cry a lot”, “I am nervous, high-strung, or tense”, and “I have trouble enjoying myself”. Response options were: “definitely not like me”, “not like me”, “somewhat like me”, “like me”, and “definitely like me”. The scale derived from these 7 items has good internal consistency (Cronbach’s alpha = 0.78) [29, 30]. We used factor analysis to derive the scale and then normalized the factor derived scale to a Z-score with a normal distribution. The one week test-re-test reliability of the internalizing symptoms Z-score in our sample was very good (ICC = 0.78).

### Covariates considered in the imputation

The imputation was informed by variables that are associated with sedentary time, or are commonly included in analyses with health indicators, in children. This included several variables related to the timing of the epochs. These timing variables were included because sedentary time differs across seasons [31], days of the week [32], and time of day [33]. Season of data collection was classified as spring (Apr – Jun), summer (Jul – Aug), fall (Sep – Nov), or winter (Dec – Mar) based on a combination of weather patterns and school calendars. Each day was classified as a school day, non-school day (i.e., weekends and holidays), or a non-school day where the child was enrolled in a day camp. The time of day was categorized for each epoch as 00:00–09:29, 09:30–14:59, 15:00–17:59, or 18:00–23:59 according to typical times for children’s school and social calendars. The interaction between type of day and time of day was also included in the imputation.

The imputation was also informed by socio-demographic and health information related to sedentary time [34, 35], including age, biological maturation (based on years from peak height velocity [36]), sex, race (white or other), family structure (dual parent household, single parent household, no response), annual family income in $CDN (≤ $50,000, $50,001 – $100,000, ≥ $100,000), parental education (high school or less, 2-y college, 4-y college/university or higher), the presence of a chronic medical condition (yes or no), the frequency of fast food consumption (rarely or never, 1 time/month, ≥ 2 times/month), and the frequency/week of snacking while engaged in screen time behaviours (continuous). The interaction between age and sex was also included in the imputation.

The imputation was also informed by four sedentary behaviour variables that were captured on a questionnaire completed by child participants: 1) average recreational screen time in hours/day, which included time spent watching TV, movies, and videos (i.e., YouTube), playing passive video games, and other recreational screen time (i.e., using a computer, tablet, or smartphone), 2) the number of hours children report doing homework (no homework, < 1 h/day, ≥1 h/day), 3) the number of electronic devices in the home (continuous), and 4) the presence of household media rules (yes or no). The one week test-re-test reliability of these questionnaire items in our sample were in the very good to excellent range (ICC = 0.74 for screen time and 0.94 for number of electronic devices, κ = 0.87 for homework and 0.85 for media rules).

### Processing of accelerometer data to derive estimates of sedentary time

Accelerometer data in 15 s epochs were concatenated and were then merged with the covariate and health indicator data described above. Data processing steps were completed on the original accelerometer dataset as well as two subsequent datasets that were created following two multiple imputation approaches (see Statistical Analysis section). Hereafter, these three datasets are referred to as: 1) nonimputed, 2) imputed dataset I, and 3) imputed dataset II. In all three datasets, the first processing step was to remove all time spent sleeping using self-reported sleep and wake times. Next, all epochs that occurred during nonwear time were identified. While there are many definitions of nonwear time [17], there is only one commonly used definition of nonwear time for the Actical accelerometer in this population. Nonwear time included both the time that children identified as having removed their accelerometer device as well as 60 min with consecutive zero counts/minute with up to two non-zero minutes [18]. There were some discrepancies between the two approaches for identifying nonwear time that usually occurred because the reported nonwear times were shorter than 60 min (e.g., removing the device for a 15 min shower), which is commonly observed [37].

In the nonimputed dataset only, all epochs that occurred during nonwear time were removed, days with < 10 h of wear time were deleted [18], and participants with < 4 days with ≥10 h of wear time were excluded from the dataset [38]. Following this, sedentary time was derived by summing epochs with < 25 counts/15 s [39, 40] for each day and a daily average was determined. Conversely, in the imputed datasets, all nonwear time was retained. Since we were ultimately interested in whether an epoch was classified as sedentary or not, as opposed to the distribution of count values, we created a dichotomous indicator variable for sedentary time. The indicator variable was coded as a 1 if the accelerometer count for that epoch was < 25 counts/15 s, as a 0 if the accelerometer count for that epoch was ≥25 counts/15 s, and as missing if the accelerometer epoch occurred during a period of nonwear time. These missing values for the dichotomous indicator variable were then imputed using multiple imputation (see Statistical Analysis section).

### Statistical analysis

The accelerometer count data were heavily skewed and zero-inflated. This unique distribution, along with available capabilities of existing statistical software, limited the imputation approach we could select. Since we were ultimately interested in deriving estimates of sedentary time (rather than the distribution of accelerometer count data per se), we simplified the imputation approach by imputing missing values for the dichotomous sedentary time indicator variable. We used a multiple imputation model that applied logistic regression (PROC MI in SAS 9.4) to the complete data and imputed the missing sedentary time indicator values (1 or 0) by simulating draws from the posterior predictive distribution of the parameters [41]. In this way, values from simulated draws for each epoch are used to determine if the sedentary time indicator value will be classified as sedentary or not, and this is informed by the likelihood of a wear time epoch being classified as sedentary or not using the parameter estimates from the logistic regression. Five iterations were performed [41, 42]. Parameter estimates obtained from each of the iterations were combined using PROC MIANALYZE in SAS 9.4 to calculate the final estimates presented herein. Within each imputation iteration, sedentary time (in minutes) for each day was determined by summing all epochs with a value of 1 for the sedentary time indicator variable (i.e., either sedentary, or missing and subsequently imputed as being sedentary) and dividing by 4. The proportion of time spent sedentary was determined by dividing this value by the total number of wear minutes during wake time. Following this, daily averages of sedentary time in minutes/day were then determined for each participant, which were subsequently used in the analyses. This resulted in five estimates (one per iteration) which were averaged to obtain an overall estimate. The differences between the estimates captured the uncertainty arising from imputing nonwear time and was incorporated in estimating variance. This same approach was applied to obtain the final estimate of the proportion of time spent sedentary.

The multiple imputation approach described above was performed twice to derive imputed datasets I and II, respectively. All variables that were in subsequent analyses, including the outcome variables, were included in the imputation models [43], which is standard practice [43–45]. This included variables related to the timing of nonwear accelerometer epochs, socio-demographic information, as well as the health indicator variables (cardiometabolic risk factor and internalizing symptoms Z-scores). Predictor variables used to derive imputed dataset II additionally included the four self-reported sedentary behaviour variables.

Means and 95% confidence intervals were used to describe sedentary time. General linear models were used to examine the association between sedentary time and both cardiometabolic risk factor and internalizing symptoms Z-scores. Covariates were the same as those included in the imputation, with the addition of mean minutes/day of moderate-to-vigorous physical activity, which was defined as ≥375 counts/15 s [39]. Parameter estimates and 95% confidence intervals were used to assess the strength of associations. Because the nonimputed and imputed datasets were derived from the same participants these data violated the assumption of independence which underpins many traditional statistical tests. Thus, we could not compare estimates of sedentary time between the nonimputed and imputed datasets using traditional statistical inference. Moreover, we could not compare the regression models between the nonimputed and imputed datasets using traditional model building statistics; the model parameters are identical, rather it is the number of participants and the method of deriving estimates of sedentary time that are different. In light of this, we compared estimates of sedentary time and regression models between the nonimputed and imputed datasets using descriptive and graphical methods.

To determine if the results varied according to accelerometer wear compliance, we conducted a sensitivity analysis wherein analyses were conducted separately in participants with 7 days with ≥10 h of wear time vs. participants with < 7 days with ≥10 h of wear time. Because few participants had only 4 or 5 days with ≥10 h of wear time, participants were not separated further. We also investigated if the results varied according to the amount of nonwear time that was imputed for each participant, where participants were grouped into tertiles based on the percentage of nonwear time (i.e., nonwear time / wear time * 100). Since this latter sensitivity analysis was not informative beyond the former, these results are not shown.

## Results

### Descriptive characteristics

Participant characteristics are in Table 1. The average age was 11.5 years and 50% of participants were boys. The majority were white (91%) and did not have a chronic medical condition (96%). Participants lived in mostly dual-parent homes (85%) and their parents reported a relatively high socio-economic status.

**Table 1:** Descriptive characteristics of study participants (*n* = 452)

Variable	N	%
Sex		
Male	225	50.2
Female	227	49.8
Age, y		
10	113	25.0
11	114	25.2
12	117	25.9
13	108	23.9
Race		
White	409	90.5
Other	44	9.5
Family structure		
Dual parent	384	85.0
Single parent	65	14.4
No response	3	0.6
Family income ($CDN per year)		
≤ $50,000	72	15.9
$50,001 – $100,000	124	27.4
> $100,000	203	44.9
No response	53	11.7
Parental education		
High school or less	41	9.0
2-y college	140	31.0
4-y college/university or higher	271	60.0
Chronic medical condition		
No	435	96.2
Yes	17	3.8
Fast food consumption		
Rarely or never	165	36.5
1 time/month	100	22.1
≥ 2 times/month	187	41.4
Snacking frequency in front of a screen		
0–4 times/week	306	67.7
5–9 times/week	95	21.0
≥ 10 times/week	51	11.3
Homework time		
No homework	144	31.9
< 1 h/day	204	45.1
≥ 1 h/day	104	23.0
Presence of household media rules		
No	133	29.4
Yes	319	70.6

### Description of accelerometer data and nonwear time

After removal of epochs that occurred during sleep periods, the accelerometer dataset included 10,989,151 epochs from 3162 days from 452 participants. A total of 2561 accelerometer nonwear periods were identified, which corresponded to 843,738 epochs. The mean (95% CI) duration of these nonwear periods was 86 min (82, 90). After removing accelerometer nonwear time and days and participants with insufficient data, the nonimputed dataset included 10,145,413 epochs from 2938 days from 442 participants. This represented 92% of total epochs collected during waking hours, 93% of total days, and 98% of participants. Of these 442 participants, 336 (76%) had 7 days with ≥10 h of wear time, while 69 (16%), 24 (5%), and 13 (3%) had 6, 5, and 4 days with ≥10 h of wear time, respectively. Among all 452 participants, the mean proportion of waking time classified as nonwear time was 8% and ranged from 0 to 48%.

On 64% of days, no nonwear periods were identified by the algorithm or recorded by participants. For algorithm-derived nonwear time, one period was observed on 26% of days, while 2 or 3+ periods were observed on 8 and 3% of days, respectively. Likewise, for recorded non-wear time, one period was observed on 29% of days, while 2 or 3+ periods were observed on 6 and 1% of days, respectively. Overall, 75% of recorded nonwear periods were shorter than 1 h. The proportion of these non-wear periods was highest during the evening hours (18:00–23:59 = 38%), relative to the other times of day – morning (00:00–9:29 = 18%), day-time (9:30–14:59 = 23%), and afternoon (15:00–17:59 = 21%). However, it should be noted that the duration of these time-of-day categories are not equivalent, which was done by design to reflect children’s school and social calendars.

### Imputation descriptive information

The parameter estimates from the logistic regression used to impute sedentary time during nonwear time are shown in Table 2. The number of variables and interactions in this model make interpreting and comparing effect sizes difficult. Therefore, to simplify interpretation we calculated the predicted probability of an epoch being classified as sedentary for each predictor variable while holding all other variables constant at their mean, and accounting for the interactions (Table 3). The strongest predictors of sedentary time for both imputed datasets included age and sex (i.e., particularly among older girls) and time and type of day (i.e., particularly during the evening hours on non-school days).

**Table 2:** Logistic regression parameter estimates used to predict sedentary time for individual nonwear epochs

	Imputed dataset I		Imputed dataset II	
	Parameter estimate (SE)	*p*-value	Parameter estimate (SE)	p-value
Intercept	0.4107 (0.0169)	<.0001	0.3787 (0.0178)	<.0001
Age, y	0.0580 (0.0013)	<.0001	0.0568 (0.0013)	<.0001
Sex				
Male	0 (referent)	–	0 (referent)	–
Female	−0.0540 (0.0075)	<.0001	−0.0442 (0.0074)	<.0001
Race				
White	0 (referent)	–	0 (referent)	–
Other	0.0146 (0.0013)	<.0001	0.0146 (0.0013)	<.0001
Family structure				
Dual parent	0 (referent)	–	0 (referent)	–
Single parent	−0.1253 (0.0035)	<.0001	−0.1258 (0.0034)	<.0001
No response	0.1843 (0.0062)	<.0001	0.2024 (0.0062)	<.0001
Family income				
≤ $50,000	0 (referent)	–	0 (referent)	–
$50,001 – $100,000	0.0390 (0.0013)	<.0001	0.0424 (0.0013)	<.0001
≥ $100,000	−0.0531 (0.0013)	<.0001	−0.0427 (0.0013)	<.0001
No response	−0.0616 (0.0019)	<.0001	−0.0644 (0.0017)	<.0001
Parental education				
High school or less	0 (referent)	–	0 (referent)	–
2-y college	0.0077 (0.0013)	<.0001	0.0055 (0.0014)	<.0001
4-y college/university or higher	−0.0352 (0.0012)	<.0001	−0.0292 (0.0013)	<.0001
Chronic medical condition				
Yes	0 (referent)	–	0 (referent)	–
No	−0.0027 (0.0019)	.1568	−0.0069 (0.0020)	.0006
Fast food consumption				
Rarely or never	0 (referent)	–	0 (referent)	–
1 time/month	−0.0303 (0.0012)	<.0001	−0.0302 (0.0012)	<.0001
≥ 2 times/month	0.0280 (0.0010)	<.0001	0.0219 (0.0010)	<.0001
Snacking frequency, times per week	−0.0035 (0.0002)	<.0001	−0.0071 (0.0002)	<.0001
Maturity (per unit of maturity offset)	0.0180 (0.0014)	<.0001	0.0151 (0.0014)	<.0001
Cardiometabolic risk Z-score	0.0643 (0.0011)	<.0001	0.0607 (0.0012)	<.0001
Internalizing symptoms Z-score	0.0355 (0.0008)	<.0001	0.0297 (0.0007)	<.0001
Season				
Spring	0 (referent)	–	0 (referent)	–
Summer	0.0138 (0.0018)	<.0001	0.0122 (0.0020)	<.0001
Fall	0.0196 (0.0012)	<.0001	0.0226 (0.0014)	<.0001
Winter	0.0552 (0.0012)	<.0001	0.0612 (0.0012)	<.0001
Type of day				
School day	0 (referent)	–	0 (referent)	–
Weekend or holiday	0.2005 (0.0022)	<.0001	0.1968 (0.0025)	<.0001
Camp day	−0.1504 (0.0042)	<.0001	−0.1433 (0.0051)	<.0001
Time of day				
00:00–09:29	0 (referent)	–	0 (referent)	–
09:30–14:59	−0.1329 (0.0032)	<.0001	−0.1339 (0.0033)	<.0001
15:00–17:59	−0.1668 (0.0037)	<.0001	−0.1664 (0.0038)	<.0001
18:00–23:59	0.1883 (0.0040)	<.0001	0.1900 (0.0040)	<.0001
Type of day x time of day interaction				
School day, all hours	0 (referent)	–	0 (referent)	–
Weekend, 00:00–09:29	0 (referent)	–	0 (referent)	–
Weekend, 09:30–14:59	−0.0644 (0.0033)	<.0001	−0.0617 (0.0035)	<.0001
Weekend, 15:00–17:59	−0.0393 (0.0039)	<.0001	−0.0394 (0.0039)	<.0001
Weekend, 18:00–23:59	−0.1034 (0.0039)	<.0001	−0.1058 (0.0042)	<.0001
Camp day, 00:00–09:29	0 (referent)	–	0 (referent)	–
Camp day, 09:30–14:59	−0.0555 (0.0062)	<.0001	−0.0593 (0.0066)	<.0001
Camp day, 15:00–17:59	0.0082 (0.0073)	.2589	0.0084 (0.0073)	.2505
Camp day, 18:00–23:59	0.0766 (0.0077)	<.0001	0.0816 (0.0079)	<.0001
Age x sex interaction				
Older males	0 (referent)	–	0 (referent)	–
Older females	0.0083 (0.0007)	<.0001	0.0081 (0.0006)	<.0001
Screen time (average hours/day)	N/A	–	0.0105 (0.0002)	<.0001
# of media devices in the home	N/A	–	0.0018 (0.0003)	<.0001
Homework time				
No homework	N/A	–	0 (referent)	–
< 1 h/day	N/A	–	−0.0229 (0.0010)	<.0001
≥1 h/day	N/A	–	0.0011 (0.0012)	.3596
Presence of household media rules				
No	N/A	–	0 (referent)	–
Yes	N/A	–	0.0073 (0.0009)	<.0001

**Table 3:** Predicted probability of epochs being imputed as sedentary while holding variables constant at their average

	Imputed dataset I	Imputed dataset II
Variable	% probability	% probability
Age and sex		
10-y old boy	72.1	72.1
10-y old girl	73.3	73.3
11-y old boy	73.3	73.3
11-y old girl	74.5	74.5
12-y old boy	74.4	74.4
12-y old girl	75.8	75.8
13-y old boy	75.5	75.5
13-y old girl	77.0	77.0
Race		
White	74.2	74.2
Other	74.5	74.5
Family structure		
Dual parent	74.6	74.6
Single parent	72.1	72.1
No response	77.9	77.9
Family income		
≤ $50,000	74.6	74.6
$50,001 – $100,000	75.4	75.4
≥ $100,000	73.6	73.6
No response	73.5	73.5
Parental education		
High school or less	74.6	74.6
2-y college	74.8	74.8
4-y college/university or higher	73.9	73.9
Chronic medical condition		
Yes	74.3	74.3
No	74.2	74.2
Fast food consumption		
Rarely or never	74.4	74.4
1 time/month	73.8	73.8
≥ 2 times/month	74.9	74.9
Snacking frequency (per SD increase vs. mean)	−0.25	− 0.52
Maturity (per SD increase vs. mean)	0.49	0.41
Cardiometabolic risk Z-score (per SD increase vs. mean)	0.89	0.83
Internalizing symptoms Z-score (per SD increase vs. mean)	0.67	0.56
Season		
Spring	73.8	73.8
Summer	74.4	74.4
Fall	74.1	74.1
Winter	74.8	74.8
Type and time of day		
School day, 00:00–09:29	73.7	73.7
School day, 09:30–14:59	71.1	71.1
School day, 15:00–17:59	70.4	70.4
School day, 18:00–23:59	77.2	77.2
Non-school day, 00:00–09:29	77.4	77.4
Non-school day, 09:30–14:59	73.8	73.8
Non-school day, 15:00–17:59	73.6	73.6
Non-school day, 18:00–23:59	78.9	78.9
Camp day, 00:00–09:29	70.7	70.7
Camp day, 09:30–14:59	66.7	66.7
Camp day, 15:00–17:59	67.3	67.3
Camp day, 18:00–23:59	75.9	75.9
Screen time (per SD increase vs. mean)	N/A	0.78
# of media devices in the home (per SD increase vs. mean)	N/A	0.09
Homework time		
No homework	N/A	74.7
< 1 h/day	N/A	74.3
≥ 1 h/day	N/A	74.7
Presence of household media rules		
No	N/A	74.4
Yes	N/A	74.6

### Estimates of sedentary time for nonimputed and imputed datasets

Mean (95% CI) sedentary time was 599 (594, 604) minutes/day in the nonimputed dataset and 632 (627, 637) minutes/day in both imputed datasets I and II (Table 4). On average, sedentary time was 33 (30, 36) minutes/day lower in the nonimputed dataset by comparison to the imputed datasets. The mean difference in sedentary time between the imputed datasets was 0 (− 4.9, 4.4) minutes/day. There was no meaningful difference in the proportion of wear time spent sedentary between the nonimputed and imputed datasets (72% vs. 73%).

**Table 4:** Sedentary time in the nonimputed and imputed datasets

Dataset	Mean (95% CI) min/day of sedentary time	Mean (95% CI) min/day of wear time	Mean (95% CI) percentage of wear time spent sedentary
Nonimputed dataset (*n* = 442)	599 (594, 604)	826 (821, 831)	72 (72, 73)
Imputed dataset I (n = 452)^a^	632 (627, 637)	868 (865, 873)	73 (72, 73)
Imputed dataset II (n = 452)^b^	632 (627, 637)	868 (865, 873)	73 (72, 73)

^a^Imputation based on sociodemographic (age, sex, maturity, race, family structure, parental education and household income), health (cardio-metabolic risk Z-score, internalizing symptoms Z-score, and presence of a chronic health condition), behavioural (frequency of fast food consumption and snacking in front of a screen), and time (time of day, type of day, and season)

^b^Imputation based on total screen time, homework time, presence of household media rules, and the number of screen-based devices in the household, in addition to the variables used in imputed dataset I

### Associations between sedentary time and health indicators

After adjusting for covariates, in the nonimputed dataset sedentary time was associated with the cardiometabolic risk factor Z-score such that each 60 min/day increase in sedentary time was associated with 0.09 unit increase in the cardiometabolic risk factor Z-score (Table 5). This association was stronger in the imputed datasets as each 60 min/day increase in sedentary time was associated with a 0.14 unit increase in the cardiometabolic risk factor Z-score. Sedentary time was not associated with the internalizing symptoms Z-score in any of the datasets.

**Table 5:** Associations between sedentary time, cardio-metabolic risk, and internalizing symptoms

	Cardio-metabolic risk Z-score		Internalizing symptoms Z-score	
	Parameter estimate (95% CI) for sedentary time	p-value	Parameter estimate (95% CI) for sedentary time	*p*-value
Nonimputed dataset	0.092 (0.007, 0.178)	0.035	0.032 (−0.087, 0.151)	0.601
Imputed dataset I^a^	0.137 (0.053, 0.221)	0.002	0.040 (−0.082, 0.162)	0.520
Imputed dataset II^b^	0.136 (0.053, 0.220)	0.002	0.040 (−0.082, 0.162)	0.522

Note: Parameter estimates indicate the change in cardio-metabolic risk or Internalizing symptoms Z-score for every 60-min increase in sedentary time. All models are adjusted for age, sex, maturity, season, race, family structure, parental education, household income, presence of a chronic health condition, fast food consumption, snacking frequency, and moderate-to-vigorous physical activity

Abbreviations: *CI* confidence interval

^a^Imputation based on sociodemographic (age, sex, maturity, race, family structure, parental education and household income), health (cardio-metabolic risk Z-score derived from body mass index, resting heart rate, and systolic blood pressure; internalizing symptoms Z-score, and presence of a chronic health condition), behavioural (frequency of fast food consumption and snacking in front of a screen), and time (time of day, type of day, and season)

^b^Imputation based on variables used in imputed dataset I as well as screen time, homework time, the number of electronic devices in the home, and the presence of household media rules

### Sensitivity analyses by compliance

**Fig. 1:**
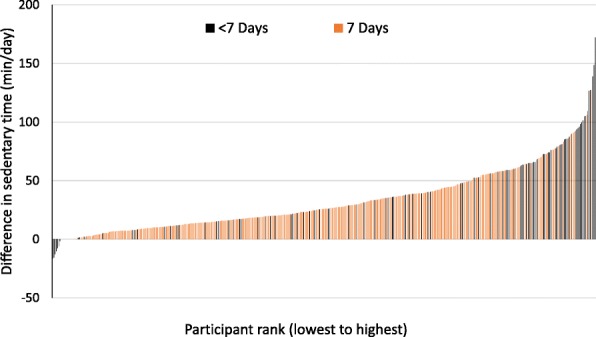
Differences in minutes/day of sedentary time between nonimputed and imputed datasets by accelerometer compliance

We considered whether the magnitude of the differences between the nonimputed dataset and imputed dataset I varied according to accelerometer wear compliance. Results are not presented for imputed dataset II because they were the same as those for imputed dataset I. As shown in Fig. 1, participants with the largest differences between the nonimputed and imputed datasets tended to have < 7 days with ≥10 h of wear time.

We also considered whether the association between sedentary time and health indicators was influenced by compliance. In those with 7 days with ≥10 h of wear time, sedentary time was not associated with the cardiometabolic risk factor Z-score in the nonimputed dataset (β = 0.089; 95%CI: -0.014, 0.193), but a positive association was observed in imputed dataset I (β = 0.111; 95%CI: 0.011, 0.212). Likewise, in those with < 7 days with ≥10 h of wear time, sedentary time was not associated with cardiometabolic risk factor Z-score in the nonimputed dataset (β = 0.072; 95%CI: -0.113, 0.256), but a positive association was observed in imputed dataset I (β = 0.189; 95%CI: 0.017, 0.361). No significant associations were observed for the internalizing symptoms Z-score.

**Fig. 2:**
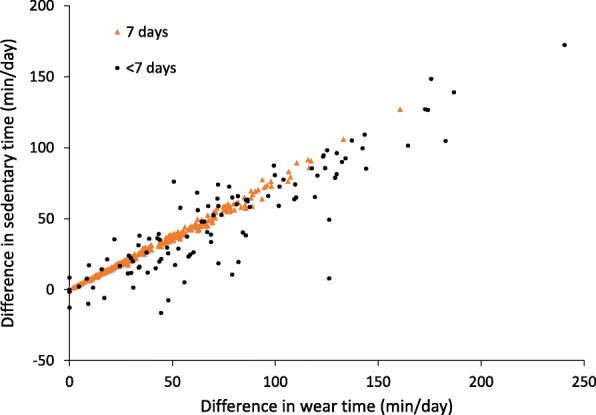
Scatterplot of sedentary time and wear time between nonimputed and imputed datasets by accelerometer compliance

Fig. 2 displays the difference in mean wear time and sedentary time between the nonimputed dataset and imputed data I according to accelerometer compliance (7 days vs. < 7 days). This figure illustrates that differences in sedentary time between the datasets were strongly correlated with the amount of nonwear time; however, this relationship was not as strong among those with < 7 days with ≥10 h of wear time (*r* = 0.89) as it was among those with 7 days with ≥10 h of wear time (*r* = 0.99).

To assist in the interpretation of the sensitivity analysis findings, we considered if days with < 10 h of wear time were different from days with ≥10 h. A higher proportion of days with < 10 h of wear time occurred on non-school days by comparison to days with ≥10 h of wear time (44% vs. 28%). A greater proportion of days with < 10 h of wear time occurred during the summer months by comparison to days with ≥10 h of wear time (38% vs. 17%). However, this was not explained by children’s participation in summer camps on weekdays (children accumulated ≥10 h of wear time on 94% of summer camp days).

## Discussion

Epochs that occur during accelerometer nonwear periods are traditionally removed during accelerometer data processing. In this study of 10–13 year old children, we used multiple imputation to impute accelerometer epochs that occurred during these nonwear periods, and determined if this influenced estimates of sedentary time and the association between sedentary time and health indicators. The imputation was informed by variables related to the timing of the accelerometer epochs, socio-demographic and health information, as well as self-reported sedentary behaviour data. Following multiple imputation, estimates of sedentary time increased by 33 min/day (5% relative increase), and the association between sedentary time and cardiometabolic risk factor Z-score was stronger (49% change in β coefficient). This suggests that removing nonwear accelerometer data leads to meaningfully lower estimates of sedentary time (in minutes/day) and biases the association between sedentary time and cardiometabolic risk towards the null.

To our knowledge, this is the first paper to examine the impact of using a multiple imputation approach to impute nonwear accelerometer epochs on sedentary time. However, previous studies have imputed accelerometer nonwear time for physical activity. We previously showed that a similar imputation approach did not meaningfully influence estimates of time spent in moderate-to-vigorous physical activity [25]. Other imputation approaches have similarly shown no or very small differences in moderate-to-vigorous physical activity [21, 46]. The discrepant finding for sedentary time in the current paper may reflect that a much larger proportion of the day, and likely nonwear periods, is spent at a sedentary rather than a moderate-to-vigorous intensity [5, 26]. Based on the imputation results from our dataset, it appears that the proportion of time spent sedentary is similar between wear and nonwear periods. This is supported by our observation that 72 and 73% of children’s time was spent sedentary in the nonimputed and imputed datasets. Thus, the underestimation of sedentary time in minutes/day with traditional data processing likely reflects that a proportional amount of sedentary time was not captured during nonwear time, and not that nonwear time was proportionally more or less sedentary than wear time. This provides support for the external validity of the imputation approach applied herein for deriving estimates of sedentary time and associations with selected health indicators.

Accelerometer compliance in this study was much higher than what is typically observed in the literature [6, 15, 16, 20]. Three quarters of children had 7 days with ≥10 h of wear time, and few participants (2%) were excluded because of insufficient wear time. This is likely the result of using a 24-h wear protocol [47], providing compensation to participants that was partially contingent on returning the accelerometer and completing the log, and maintaining frequent contact between research staff and participants (e.g., daily text or e-mail reminders to wear the device and complete their log). Nonetheless, we showed that the effect of imputing accelerometer nonwear time on estimates of sedentary time, and of associations between sedentary time and cardiometabolic risk, was related to accelerometer compliance. Among participants who had 7 days with ≥10 h of wear time, differences in estimates of sedentary time between the nonimputed and imputed datasets were precisely associated with the amount of accelerometer nonwear time; however, this precision was considerably lower among those who had < 7 days with ≥10 h of wear time. Similarly, the difference in the estimates of association between sedentary time and cardiometabolic risk between the imputations was much greater (over 2-fold difference) among those participants who had < 7 days (vs 7 days) with ≥10 h of wear time. Therefore, in studies with lower (i.e., more typical) compliance, the impact of accelerometer nonwear time on sedentary time may be greater than that observed in the full sample of children in the current study.

In our study, children were more likely to accumulate < 10 h of wear time on days when their school-day routine is disrupted, such as on weekend days, holidays, and during the summer school break (but not when participating in a structured summer day camp). Sedentary time is typically higher on non-school days than school days [32], so excluding more non-school days would result in an underestimation of sedentary time. This helps to explain why estimates of sedentary time were higher in the imputed datasets. Moreover, children likely engage in a wider range of activities outside of their usual routine on non-school days vs. school-days. This may explain, at least in part, why differences in estimates of sedentary time before and after imputation were less precise on days when children had < 10 h of wear time.

There are two implications of our research. First, we showed that imputing accelerometer nonwear time strengthened the association between sedentary time and cardiometabolic risk, and it is possible that the null associations frequently observed in the literature [1–4] represent an under-estimation of this association as a result of accelerometer nonwear time. Going forward, researchers should consider using multiple imputation as a means to mitigate this potential bias. Second, studies analyzing time-constrained accelerometer data using compositional analysis rely on having data from a complete 24-h day [48–50]. Thus, estimates of movement behaviours (including sedentary time) are normalized to a full 24-h day [51]. Our results suggest that normalizing data to the full 24-h day would only provide reasonably valid and precise estimates of sedentary time among participants with excellent compliance (i.e., 7 out of 7 days with ≥10 h of wear time). However, this would likely bias associations between sedentary time and cardiometabolic risk towards the null, particularly for participants with lots of nonwear time. Researchers analyzing 24-h movement behaviour data should consider using multiple imputation to handle accelerometer nonwear time. However, at present the current approach is limited to estimates of individual behaviours, and future work should expand this approach to include multiple movement behaviours.

This study is not without limitation. First, the limited amount of nonwear time in the current study (normally regarded as a strength) precluded us from examining the effect of multiple imputation by groups of participants with and without sufficient wear time Second, this imputation model did not account for the multilevel nature of the data. Third, this approach only allows for imputation of sedentary time as a binary variable. Different predictors should be used when applying this approach to other intensity categories. Many studies capture some of these predictor variables, but other variables may not typically be available. Finally, this study did not use count values as a reference for evaluating the imputation model. However, the utility of a similar imputation approach has been demonstrated by simulating missing accelerometer epochs and comparing imputed values against observed count values [24].

## Conclusion

We applied a multiple imputation approach that relied on time-based, socio-demographic, and health information to impute accelerometer epochs that occurred during periods of nonwear time. This resulted in a higher estimate of sedentary time and strengthened the association between sedentary time and cardiometabolic risk, compared with the traditional approach of deleting accelerometer nonwear time. Researchers should consider using imputation techniques rather than deleting nonwear time when deriving estimates of sedentary time in children.
